# The humanitarian crisis in Ukraine. Nurses around the world can and should unite to help

**DOI:** 10.1590/1518-8345.0000.3660

**Published:** 2022-07-08

**Authors:** Nancy Reynolds

**Affiliations:** 1 Johns Hopkins University, School of Nursing, Baltimore, MD, Estados Unidos da América

Russia’s invasion of Ukraine shows little sign of abating. In the midst of the ongoing attack on Ukraine and a growing human rights and humanitarian crisis, the nursing community must lend support.

For over two months now, Russia has launched a war on Ukraine. The conflict in Ukraine has caused civilian casualties and destruction of the civilian infrastructure. It has forced people to flee their homes seeking safety, protection and assistance. As of April 25^th^, it is estimated that over 5.2 million Ukrainians have fled their homeland to neighboring countries including Poland, Hungary, Romania, Slovakia, and Moldova^1^. It is estimated that even more have been displaced from their homes but remain in Ukraine. Conflict, and especially the Russian bombardment of civilian institutions and inability of civilians to flee in safety, has created an overwhelming internal crisis, exacerbating an already dire situation. Supply chains have been severely disrupted. Many distributors are not operational, some stockpiles are inaccessible due to military operations, medicine supplies have run low, and hospitals struggle to provide care to the sick and wounded^2^.

Nurses and other health care providers on the ground have shown remarkable dedication and bravery. When the invasion started, many helped move patients to basements for safety and stayed there caring for them for days without relief. Nurses initially had to ventilate patients manually in hospital basements and bomb shelters the United Nations wrote in a release^3^. Moreover, many have had to provide care as their health care institutions were targeted. On April 10^th^, 2022, the World Health Organization reported that it had verified more than 103 attacks on hospitals and clinics in Ukraine since the start of the invasion. The attacks so far have claimed 73 lives and injured 51^4^. Further, when people are prevented from seeking and accessing health care, either because the facilities have been destroyed or out of fear that they may become a target, the psychological stress is intense. Ukrainian civilians and health care workers alike are grappling with a multitude of emotions at once including anxiety, fear, grief, anger, depression and despair. The mental health toll wreaked by the war is vast with likely many long term ramifications^5^. 

As the war broke out, the effects of Russia’s invasion of Ukraine reverberated throughout the global nursing community. There are really no words to adequately express the scope of the shock, pain, and trauma being inflicted. Politics are often far from a central concern of nurses and armed conflicts are antithetical to the main purpose of the profession. Yet, nursing exists to satisfy a fundamental need of humanity. Without an end in sight to this and other conflicts, nurses should be involved. Certainly, none of us in a similar situation would want our global colleagues around the world to look away. Fulfilling the role of patient and public advocate dictates that nurses support actions that are in the best interest of public health. This responsibility is not limited to the workplace; it extends to the global community.

What can nurses do to lend support or show they stand in solidarity with nurses and other health care workers in Ukraine?

1. Volunteer as a nurse in Ukraine and neighboring countries. Immediate health care support is needed to prevent the country from spiraling even deeper into a humanitarian crisis that could impact tens of millions of people. Nurses are in the position to help this way can reach out to organizations seeking volunteers and offer their services. Examples include: Team Rubicom Disaster Response [International Team (http://www.teamrubiconusa.org)]; Children of War [Show Your Support - Children of War Foundation (http://www.cowf.org)]; devex [VOLUNTEER, NURSE | Devex (https://www.devex.com/jobs/volunteer-nurse-969760)]
2. In a different approach, the Johns Hopkins School of Nursing has attempted to provide psychological/mental health support from afar. It has prepared videos and compiled resources for psychosocial aid for our partner institution in Ukraine. [Psychosocial Resources - Ukraine (http://www.jhu.edu)].
3. Advocate for peace and for the protection of health care workers. Health workers throughout the country are risking their lives to serve those in need of medical services, and they, and their patients, must never be targeted. As one of the most trusted professions, nurses can draw attention to humanitarian needs and call for an end to the violence. You can sign a petition condemning the Ukraine invasion and military attacks on nurses and other health care workers [Sign petition: #NURSESFORPEACE (https://www.gopetition.com/petitions/nursesforpeace.html)] or advocate for peace by using the hashtag #NursesforPeace on social media. See the International Council of Nurses statement here: https://www.icn.ch/system/files/documents/2022-03/Statement_ICN_EFN_EFNNMA_1.pdf.
4. Donate. Donating money to organizations on the ground most familiar with the circumstances is highly valuable. Donate funds (as opposed to goods) to reputable organizations to support on the ground efforts. Teams on the ground and familiar with the cultural context are best positioned for a coordinated response to greatest extent possible. Examples include: The ICN Humanitarian Fund (https://icn-shop.myshopify.com/); Médecins San Frontières/Doctors Without Borders (https://donate.doctorswithoutborders.org/secure/donate); The World Central Kitchen [Donate to World Central Kitchen (http://www.wck.org)]; International Relief Teams [Crisis in Ukraine | Donate - International Relief Teams (http://www.irteams.org)]
5. Think long term and support refugees. The United Nations is warning that continued conflict in Ukraine could displace 10 million people - a staggering number that would make it one of the largest refugee crises on earth. Many persons will be displaced for years to come. Some nurses themselves will become refugees and displaced from their source of livelihood. They will need support to become reestablished if not able to return to their home country. The adjustment socially, economically and psychologically can be exceedingly challenging. Nurses can offer compassion and aid to refugees in their efforts to adjust. Nurses must show leadership in lending empathy to refugees whether from Ukraine or elsewhere. Contact your local UNHCR (The UN Refugee Agency) or donate International Refugee Committee (https://help.rescue.org/donate-br).

Nurses in the Ukraine will be pushed to the limit in caring for the wounded and sick. Nurses around the world must unite and stand in solidarity with nurses in Ukraine. Nurses must send an important message to the world about the values it stands for and to Ukrainian nurses that they are not alone. In times of humanitarian crises like this, acts of compassion and generosity can make an important difference.
